# Supplementary material for: Green moisture-electric generator based on supramolecular hydrogel with tens of milliamp electricity toward practical applications
Source: Nat Commun. 2024 Apr 18;15:3329. doi: 10.1038/s41467-024-47652-3 (PMC11026426; doi:10.1038/s41467-024-47652-3)
Supplement: Supplementary file 1 — Supplementary Information [file 41467_2024_47652_MOESM1_ESM.pdf]

Supplementary Information

Green moisture-electric generator based on supramolecular hydrogel with tens of milliamp electricity toward practical applications

Su Yang[1,2], Lei Zhang[3], Jianfeng Mao[4], Jianmiao Guo[4], Yang Chai[4], Jianhua Hao[4], Wei Chen[5], Xiaoming Tao[1,2*]

1 Research Institute for Intelligent Wearable Systems, The Hong Kong Polytechnic University, Hong Kong 999077, P. R. China.

2 School of Fashion and Textiles, The Hong Kong Polytechnic University, Hong Kong 999077, P. R. China.

3 Department of Materials Science and Engineering, University of Science and Technology of China, CN 230026, P. R. China.

4 Department of Applied Physics, The Hong Kong Polytechnic University, Hong Kong 999077, P. R. China.

5 National & Local Joint Engineering Research Center for Textile Fiber Materials and Processing Technology, School of Materials Science and Engineering, Zhejiang Sci-Tech University, Hangzhou 310018, P. R. China

* Corresponding author. Email: xiao-ming.tao@polyu.edu.hk

Supplementary Figures

Supplementary Figure 1. FTIR spectrum of pure AlgNa, and PVA-AlgNa based hydrogels.
Supplementary Figure 2. The V_{oc} and I_{sc} of MEG with or without PEDOT:PSS pre-coated on bottom electrode at 22 °C and 80% RH.
Supplementary Figure 3. The current curve of MEG over 1 month with the loading resistor of 1kΩ under 70% RH. The inserts shows the current curve of the MEG device at different time slots.
Supplementary Figure 4. (a) Moisture-absorption isotherm and current output of PVA-AlgNa based hydrogel vs RH variation. (b)The specific absorption kinetics curve under 10% RH of PVA-AlgNa hydrogel. The test condition is at 25 °C.
Supplementary Figure 5. (a) V_{oc} of MEG unit under different relative humidity (10-90% RH) at 22 °C. (b) V_{oc} of MEG unit under different temperature from -25 to 65 °C at 80% RH. The size of one single MEG unit is 1 cm^2, unless otherwise stated.
Supplementary Figure 6. (a) The water contents of MEGs dried at 65% and 20% RH, respectively. The temperature is fixed at 25 °C. (b) The water contents of MEGs dried at 25 °C, 50 °C, 70 °C, respectively. The RH is fixed at 20%. (c) The V_{oc} and I_{sc} for 20% and 65% RH dried MEGs, respectively. (d) The V_{oc} and I_{sc} of MEG dried at 25, 50, 70 °C, respectively. The test condition is 80% RH and room temperature.
Supplementary Figure 7. The electric generation performance of the MEG assembled by different inert electrodes. The I_{sc} output of the MEG with top electrode replacing by C (a) or Pt electrode (b) or Au electrode (c) under 80% RH. (d) V_{oc} output of the MEGs with different electrodes at the same condition. (e) The I_{sc} and V_{oc} of MEG with C-C electrodes at different RHs tested at room temperature. (f) The I_{sc} and V_{oc} of MEG with C-C electrodes at different temperature tested at 80% RH.
Supplementary Figure 8. The V_{oc} (a) and I_{sc} (b) output of MEG tested at 80 % RH in a N_2 environment.
Supplementary Figure 9. The electric generation performance of MEGs with different electrode structure (top electrode-bottom electrode: Al-C, C-Al and C-C were tested at 80% RH.
Supplementary Figure 10. (a) The I_{sc} curves with the time for different sizes including 0.01, 0.25, 1, 4, 9 cm^2. (b) I_{sc} and V_{oc} of MEG units with different $CaCl_2$ concentration from 0 to 4.8 wt% under 80% RH. (c) The V_{oc} and J_{sc} plotted against film thickness for a device size of 0.25 cm^2. The test condition is at 80% RH.
Supplementary Figure 11. Current density cycles of one MEG in response to the intermittent and periodic RH variation from 30% to 60%.
Supplementary Figure 12. (a) Cyclic electric performance of MEG with load resistance of 1 kΩ in water adsorption-dehydration-adsorption cycles under 70% RH and room temperature.

Supplementary Figure 13. (a) The Nyquist plot of MEGs at 80% RH with fitting plot. (b) The Nyquist plot of MEGs at 30% RH with fitting plot.

Supplementary Figure 14. Moisture uptake capability and current output of MEG versus time synchronously. (a) The mass increase of MEG by moisture adsorption versus time under 80% RH and room temperature. (b) The concurrent current output of MEG versus time at the same condition. (c-e) The tested current output of MEG at different time slot.

Supplementary Figure 15. (a, c) The V_{oc} curves and current output of MEG devices based on PVA-AlgNa without $CaCl_2$ over time under an open environment, respectively. (b, d) The V_{oc} curves and current output of MEG devices based on PVA with 1 kΩ external resistor under the evolution of time at 70% RH and room temperature, respectively. The insert shows the circuit diagram of MEG with one resistor.

Supplementary Figure 16. Raman spectrum of the PVA-AlgNa based hydrogel.

Supplementary Figure 17. 2D Raman mapping. (a) The moisture capturing by top surface of PVA-AlgNa based hydrogel after exposing in air for 45 min. (b) The moisture capturing by bottom surface of PVA-AlgNa based hydrogel with the same exposure time. (c) The moisture capturing by top surface of PVA after exposed in air for 45 min.

Supplementary Figure 18. Cross-sectional 2D Raman mapping of PVA-AlgNa based hydrogel with the depth after one week.

Supplementary Figure 19. In-situ FTIR spectrum tracking once the samples are exposed in the atmosphere (65% RH) versus time for PVA (a) and PVA-AlgNa based hydrogel (b).

Supplementary Figure 20. The integrated intensities of the three decoupled peaks versus time, a, 3540 cm^{-1}; b, 3115 cm^{-1}, c, 3304 cm^{-1}).

Supplementary Figure 21. Chemical component characterization of detective Na^+ ions (a) and Cl^- ions (b) variation between the two surfaces of MEG in different states. The Δc(ion) is defined as the ion element content at the top surface minus the content at the bottom surface of MEG. (c) Interaction region indicator maps and the corresponding interaction energies (kj/mol) of AlgCa-H_2O, AlgNa-H_2O, Alginate acid-H_2O. The sign$(\lambda_2)\rho$ is mapped on the isosurfaces.

Supplementary Figure 22. The ion distribution between top and bottom surface for MEG suffering from 5 hours' and 15 days' short-circuit treatment. (a) Ca element. (b) Cl element. (c) Na element.

Supplementary Figure 23. Schematic plot of the setup for the KPFM test. A piece of hydrogel absorbs moisture from its bottom side and the upper side is covered without wetting. The KPFM probe measures the nonwetting side with the time.

Supplementary Figure 24. Schematic plot of proposed working mechanism of MEG. (a) The initial state. Ions pair are distributed along the thickness direction due to the weak internal electric field of asymmetrical structure at the initial state. (b) Open voltage stage. After absorbing water molecules from air, positive ions transport from the top to the bottom side, resulting in a large open-circuit voltage. (c) Short circuit stage. The reverse migration of ions is observed with short-circuit treatment.

Supplementary Figure 25. The fabrication process diagram of large-scale integration of MEG devices. (a) Preparation of PI substrate. (b) Laser printing of bottom electrodes with optimized laser power and writing speed. (c) Stencil printing of PEDOT: PSS ink on bottom electrode as carrier transport-assisting layer. (d) Dripping well-prepared hydrogel sequentially. (e) Placing and adhering top electrodes on the top of the hydrogel by end-to-end connection. The inset is the structure of enlarged 2-serial MEG units. The details can be found in the experimental section.
Supplementary Figure 26. Scheme of large-scale integration of parallel MEG bank.
Supplementary Figure 27. The current density for the large-scale MEG array.

Supplementary Tables

Supplementary Table 1. Surface area in each ESP range on the vdW surface for PVA.
Supplementary Table 2. Surface area in each ESP range on the vdW surface for AlgNa.
Supplementary Table 3. Surface area in each ESP range on the vdW surface for AlgCa.
Supplementary Table 4. The performance comparison of current moisture electric generators.

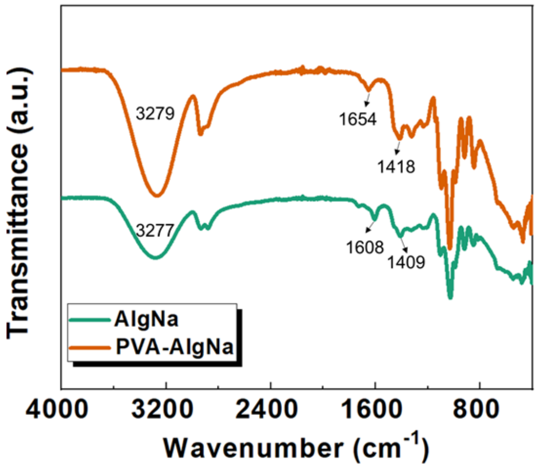

Supplementary Figure 1. FTIR spectrum of pure AlgNa, and PVA-AlgNa based hydrogels.

As shown in Supplementary Figure 1, FTIR spectrum of pure AlgNa shows the bands around 3277, 1608, and 1409 cm^{-1}, corresponding to the stretching of −OH, −COO− (asymmetric), and −COO− (symmetric) group, respectively. Compared with pure AlgNa, an obvious shift of −COO− stretching bands to higher wavenumber 1654 cm^{-1} is observed in PVA-AlgNa based hydrogel, indicating the crosslinking Ca^{2+} with −COO− of AlgNa. [1]

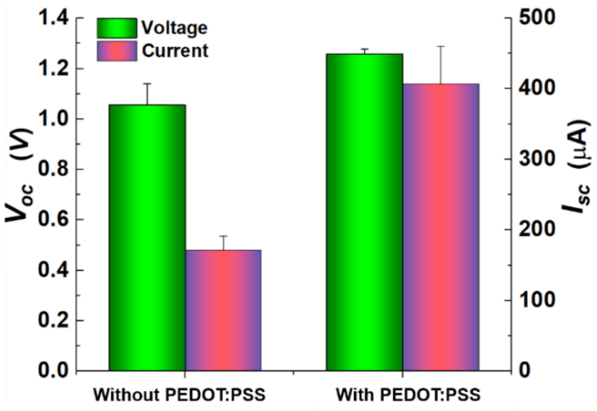

Supplementary Figure 2. The V_{oc} and I_{sc} of MEG with or without PEDOT:PSS pre-coated on bottom electrode at 22 °C and 80% RH. Data represent the mean ± standard deviation (n = 4).

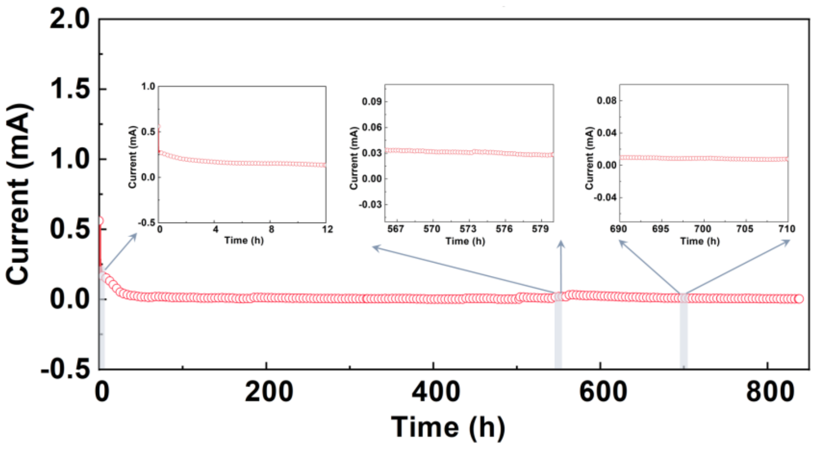

Supplementary Figure 3. The current curve of MEG over 1 month with the loading resistor of 1 kΩ under 70% RH. The inserts shows the current curves of the MEG device at different time slots.

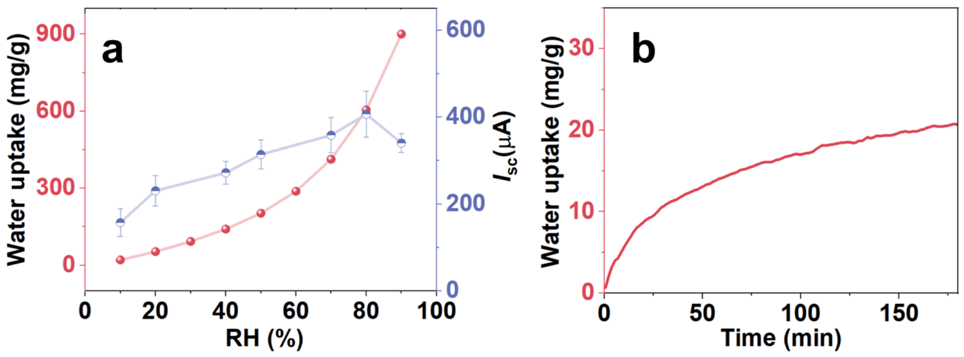

Supplementary Figure 4. (a) Moisture-absorption isotherm and current output of PVA-AlgNa based hydrogel vs RH variation. Data represent the mean ± standard deviation (n = 4). (b) The specific absorption kinetics curve under 10% RH of PVA-AlgCa/Na hydrogel. The test condition is at 25 °C.

Supplementary Figure 4 depicts the relationship of the current performance and moisture uptake capability with the RH variation. With the increase of RH from 10 to 80%, the average current augments monotonically up to 407 μA, along with a gradual enhanced moisture uptake capability. The simultaneous escalated current and moisture uptake hint the essential role of moisture in power generation. A slight decrease of current is observed at 90% RH possibly due to reduced water gradient with excessive moisture absorption. The absorption kinetics curve of MEG (Supplementary Figure 4b) shows a gradual weight increment of about 2.1 wt% after exposing in the air at 10% RH of over 180 min, demonstrating a strong water absorption capability of MEG even at harsh environment.

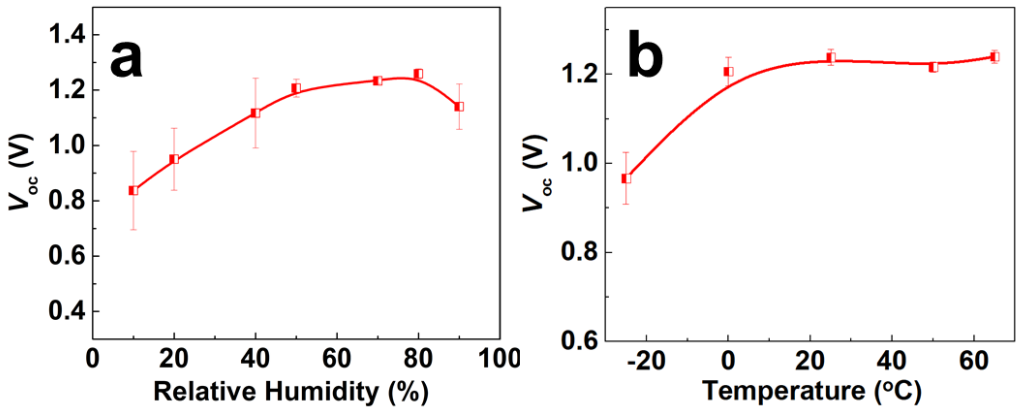

Supplementary Figure 5. (a) V_{oc} of MEG unit under different relative humidity (10-90% RH) at 22 °C. (b) V_{oc} of MEG unit under different temperature from -25 to 65 °C at 80% RH. The size of one single MEG unit is 1 cm^2, unless otherwise stated. Data represent the mean ± standard deviation (n = 4).

Fig S5a shows that the voltage outputs are gradually improved from ca. 0.8 V to 1.3 V with increasing RH to 80%. As we know, the voltage output is closely related to water gradient, or an ion concentration gradient. It is proposed that the increased RH promotes more ions dissociation and forms a larger ion concentration difference, positively enhancing voltage output. When the RH reaches 90%, the voltage slightly decreases, probably due to the inferior moisture gradient by saturation. Similar trend is observed for voltage change with the rising of temperature (Fig S5b), mainly deriving from triggered ion dissociation and diffusion. The rising of temperature promotes ion dissociation to improve ion concentration difference meanwhile accelerates the ion diffusion rate, boosting a high V_{oc} of about 1.3 V and a large current. At a low temperature of −25 °C, there still exists a voltage of ~0.9 V, demonstrating a wide environmental adaptability of MEG.

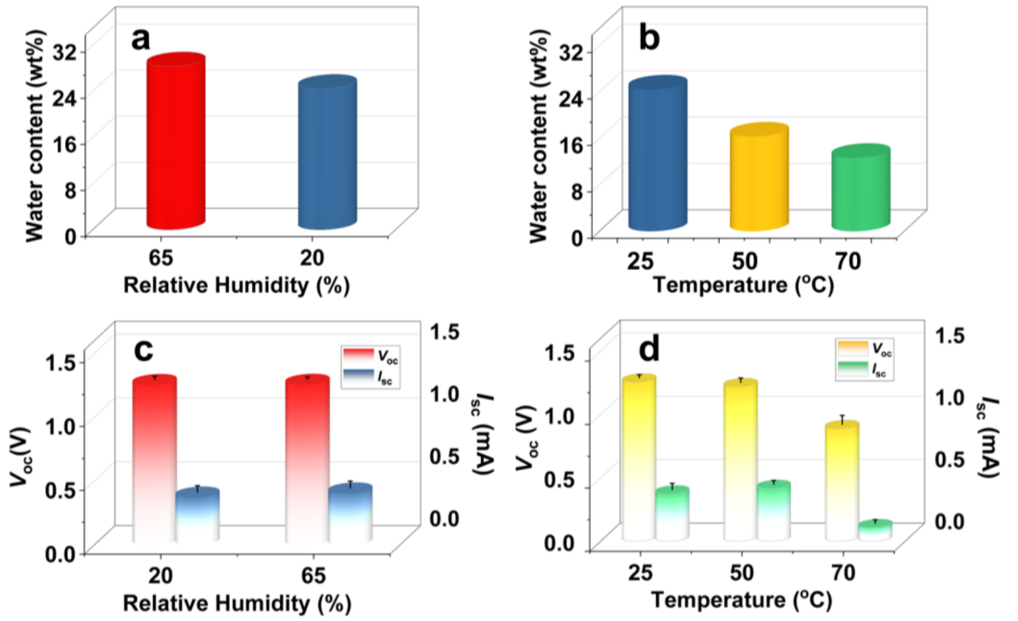

Supplementary Figure 6. (a) The water contents of MEGs dried at 65% and 20% RH, respectively. The temperature is fixed at 25 °C. (b) The water contents of MEGs dried at 25 °C, 50 °C, 70 °C, respectively. The RH is fixed at 20%. (c) The V_{oc} and I_{sc} for 20% and 65% RH dried MEGs, respectively. (d) The V_{oc} and I_{sc} of MEG dried at 25, 50, 70 °C, respectively. The test condition is 80% RH and room temperature. Data represent the mean ± standard deviation (n = 4).

The water content of hybrid hydrogel can be controlled by drying conditions, including changing RH and temperature. After 24 hours' gelation, the hybrid hydrogels went through another 12 hours' drying. For the first drying method, the samples were dried at different RHs (65% and 20%) at room temperature. As shown in Supplementary Figure 6a, the water content is about 28 wt% when samples are dried at 65% RH. In comparison, the calculated water content is about 24 wt% when the drying condition is 20% RH at room temperature, which is slightly smaller than that dried at 65%. Secondly, different temperatures (25, 50, 70 °C) with a fixed 20% RH have been employed to dry samples. Supplementary Figure 6b displays that a gradual decrease of water content is observed from 24 wt% to 13 wt% with temperature rising from 25 to 70 °C.

Furthermore, the electric outputs were tested for above samples once exposed at 80% RH and room temperature as shown in Supplementary Figure 6c. Supplementary Figure 6c shows that MEG dried at 65% delivers a high V_{oc} of ca. 1.3 V and a large I_{sc} of ca. 0.4 mA, which keeps

almost the same for MEG dried at 20%. Besides, the V_{oc} and I_{sc} of MEGs dried at 50 °C are competitive with those of MEGs dried at 25 °C, while is obviously larger than those of MEGs dried at 70 °C (Supplementary Figure 6d). The high temperature of 70 °C may cause excessive water loss (Supplementary Figure 6b) and contact issue between hydrogel and electrode, leading to a lower electric output. Based on above results, it is reasonable to deduce that the drying conditions at a wide range of 20-65% RH and 25-50 °C exert little impact on the final electric output of MEG despite the initial water content varies from 28 wt% to 16 wt%. For the underlying reason, strong moisture absorption capability of MEG guarantees that subsequent water capturing is not impacted at an unsaturated state. Thus the final electric output is also not affected when dried under suitable conditions.

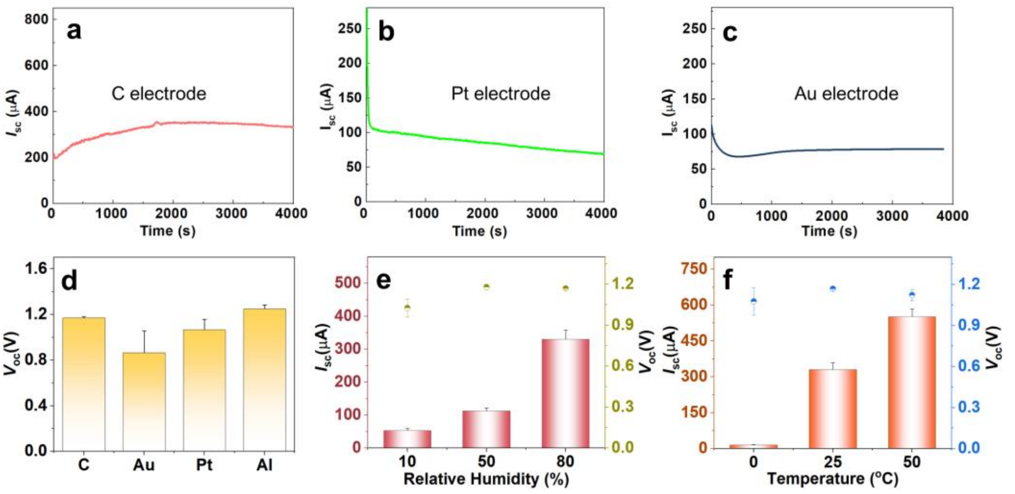

Supplementary Figure 7. The electric generation performance of the MEG assembled by different inert electrodes. The I_{sc} output of the MEG with top electrode replacing by C (a) or Pt electrode (b) or Au electrode (c) under 80% RH. (d) V_{oc} output of the MEGs with different electrodes at the same condition. (e) The I_{sc} and V_{oc} of MEG with C-C electrodes at different RHs tested at room temperature. (f) The I_{sc} and V_{oc} of MEG with C-C electrodes at different temperatures tested at 80% RH. Data represent the mean ± standard deviation (n = 4).

The results show that all MEGs with different inert electrodes yield favorable electric outputs, which are comparable with that of MEG using Al electrode. For MEG using C electrode, a continuous I_{sc} output of about 350 μA is maintained beyond 4000s at 80% RH as shown in Supplementary Figure 7a, approaching that of MEG using Al electrode. Supplementary Figure 7b and c show that MEGs using Pt and Au electrode can also deliver about 100 and 80 μA over 4000s, respectively. Such small difference for MEGs with different electrodes is acceptable

and also observed in other references.[2-5] Supplementary Figure 7d demonstrates that MEGs using other inert electrodes output similar V_{oc} with MEG using Al electrode. Based on the comparison of MEGs with different electrodes, it can be safely concluded that the supramolecular hydrogel is the key for electric generation of our MEG instead of electrodes. The supramolecular hydrogel enhances the moisture absorption of the MEG to promote the sufficient chemical conversion energy, consequently inducing high power output. Similar with other previous works, Al electrode is actually a common selection for MEGs.[3, 6] Compared to the inert electrodes like Au, Al is highly flexible, lightweight, easily accessible and fairly cheap, which is desirable for scalable and low-cost MEGs towards wide applications.

Furthermore, MEGs with C-C electrode were prepared to systematically measure the humidity and temperature-dependent characteristics. Supplementary Figure 7e shows that the average I_{sc} augments monotonically from ca. 50 μA up to ca. 330 μA with the rising of RH from 10 to 80%. The accelerated current mainly derives from enhanced moisture absorption to trigger substantial ion diffusion. Besides, V_{oc} presents a gradual rise to ~ 1.2 V with the RH. In addition, our MEG also shows great adaptability under wide range of temperature. From 0 to 50 ºC, the average J_{sc} shows a sharp climb from ca. 15 to 552 μA (Supplementary Figure 7f). Such obvious growth benefits from the synergistic effect of accelerated ion transport rate and ion concentration at high temperature. The elevated temperature also enhances V_{oc} to about 1.2 V. The humidity and temperature-dependent characteristics of MEGs with C-C electrode display the same tendency with that of C-Al electrode based MEG. Overall, MEGs with inert electrodes demonstrate comparable electric output to MEGs with C-Al electrode, even changing humidity/temperature conditions. It further verifies moisture-induced ions diffusion is the driving force for electric generation of our MEG device.

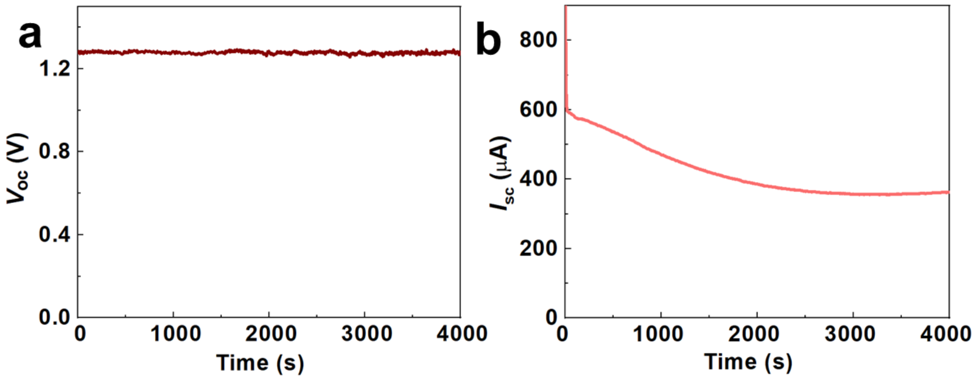

Supplementary Figure 8. The V_{oc} (a) and I_{sc} (b) output of MEG tested at 80 % RH in a N_2 environment.

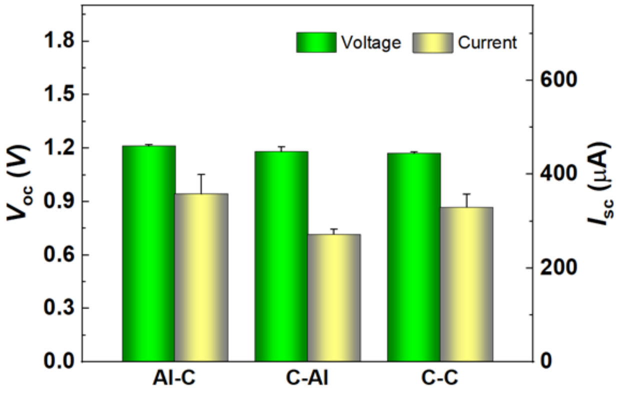

Supplementary Figure 9. The electric generation performance of MEGs with different electrode structure (top electrode-bottom electrode: Al-C, C-Al and C-C) were tested at 80% RH. Data represent the mean ± standard deviation (n = 4).

Supplementary Figure 9 shows that the MEGs with different electrode structures show similar V_{oc} output and comparable I_{sc} output, indicating relative electrode positions exert limited influence on electricity generation. In fact, In fact, asymmetric electrode design, with C as bottom electrode and multi-hole Al film as top electrode, are chosen based on the following reasons. Firstly, the flexible bottom C electrodes can be fabricated by directly laser patterning on soft PI substrate efficiently and precisely on a large scale. [7, 8] It is easy to realize the localization and scale-up for large integration of MEG arrays. Secondly, flexible and lightweight Al films can easily connect to adjacent C electrodes. Besides, Al film is highly conductive, easily accessible and fairly cheap, which is desirable for scalable and low-cost MEGs towards wide applications. The conductivity of Al film is not influenced by punching

holes. In contrast, if asymmetric electrode with top electrode (C)-bottom electrode (Al) structure is adopted, it needs extra substrate to load Al films as bottom electrodes. Moreover, the conductivity of C electrodes will be impaired by punching holes process, which may result in slightly decreased current output as shown in Supplementary Figure 9. Thus, we give preference to asymmetric electrodes with Al-C structure.

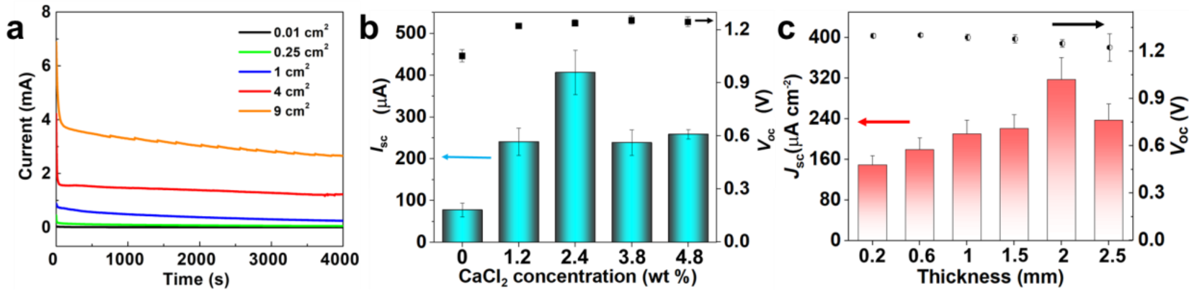

Supplementary Figure 10. (a) The I_{sc} curves with the time for different sizes including 0.01, 0.25, 1, 4, 9 cm^2. (b) I_{sc} and V_{oc} of MEG units with different $CaCl_2$ concentration from 0 to 4.8 wt% under 80% RH. (c) The V_{oc} and J_{sc} plotted against film thickness for a device size of 0.25 cm^2. The test condition is at 80% RH and room temperature. Data represent the mean ± standard deviation (n = 4).

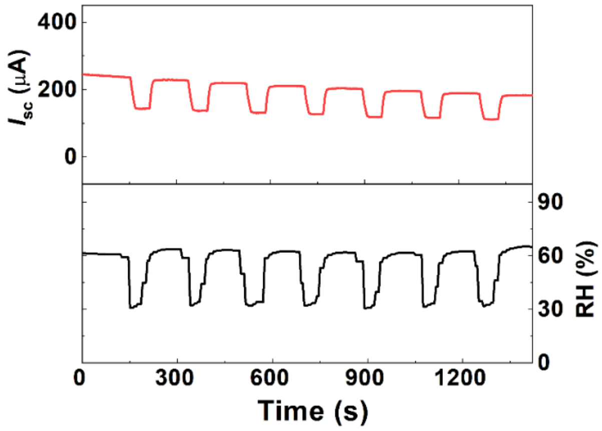

Supplementary Figure 11. Current density cycles of one MEG in response to the intermittent and periodic RH variation from 30% to 60%.

To quantitatively measure the response to the environmental humidity, the current output of MEG is synchronically tested with the cycling variation of RH. The result is shown in Supplementary Figure 11. When RH decreases from 60% to 30%, the short-circuit current (I_{sc}) rapidly drops from ca. 245 μA to ca. 145 μA. Then the I_{sc} remains for 1 min with RH holding

at 30%. Subsequently, I_{sc} immediately rises up accompanied by fast hydration. The regular change of current output of MEG is well observed when response to the cycling variation of RH, suggesting the power generation is closely related to the hydration process.

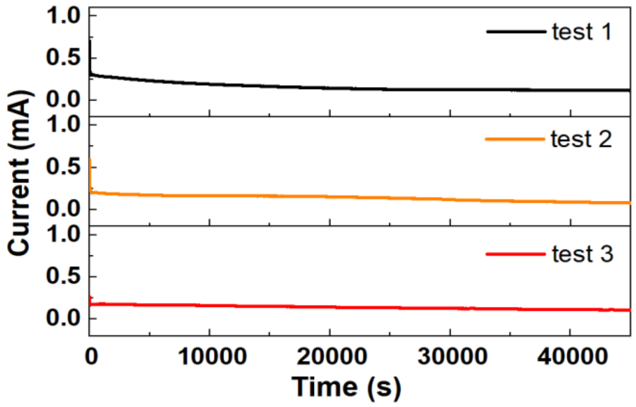

Supplementary Figure 12. (a) Cyclic electric performance of MEG with load resistance of 1 kΩ in water adsorption-dehydration-adsorption cycles under 70% RH and room temperature.

To test the cycle electric performance, MEG goes through water adsorption-dehydration-adsorption cycles. The resulting current curves are demonstrated in Supplementary Figure 12a. The first absorption cycle enables MEG deliver a sustained current of about 0.22 mA. After that, MEG undergoes dehydration process by drying at 20% RH and room temperature about 24 h. The subsequent absorption cycles also drive MEG to generate sustained current output despite the current outputs become slightly smaller, displaying decent cycle electric performance of MEG.

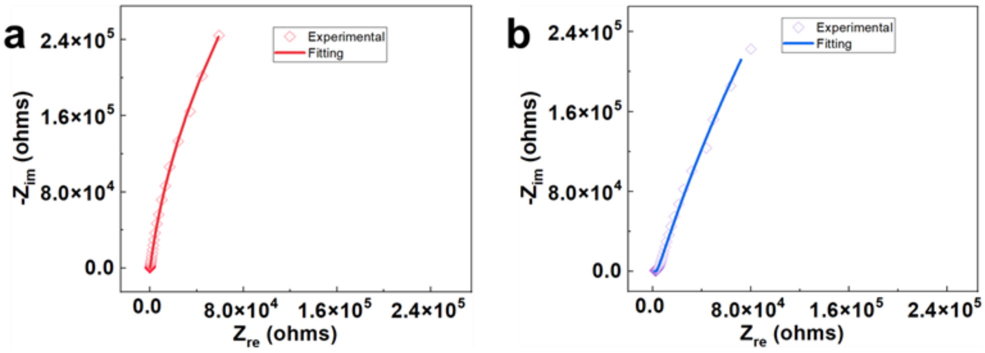

Supplementary Figure 13. (a) The Nyquist plot of MEGs at 80% RH with fitting plot. (b) The Nyquist plot of MEGs at 30% RH with fitting plot.

To investigate the effect of RH on the internal resistance (R_i), electrochemical impedance spectra (EIS) of MEGs at 30% and 80% RH were measured to calculate the ionic conductivity (δ_{dc}), deduced by the equation[9]: $\delta_{dc} = (\frac{1}{R_i})(\frac{t}{A})$, where t is the hydrogel's thickness, and A is the hydrogel's surface area. Here R_i represents the overall internal resistance of MEG device.[10] Supplementary Figure 13a and b display the simulation results of Nyquist plots of MEGs at 30% and 80% RH. The calculated δ_{dc} for MEGs at 30% RH and 80% RH are $7.46*10^{-5}$ S/cm and $1.02*10^{-3}$ S/cm, respectively. That means the ionic conductivity enhances with the RH increase. Furthermore, a decreased slope for MEG at 30% RH indicates that the charging process was limited by ion diffusion in the hydrogel. [11] From both above results, it is inferred that the increase in ambient humidity can synergistically reduce the internal resistance of MEG device and trigger the ion diffusion, resulting in a soaring current output.

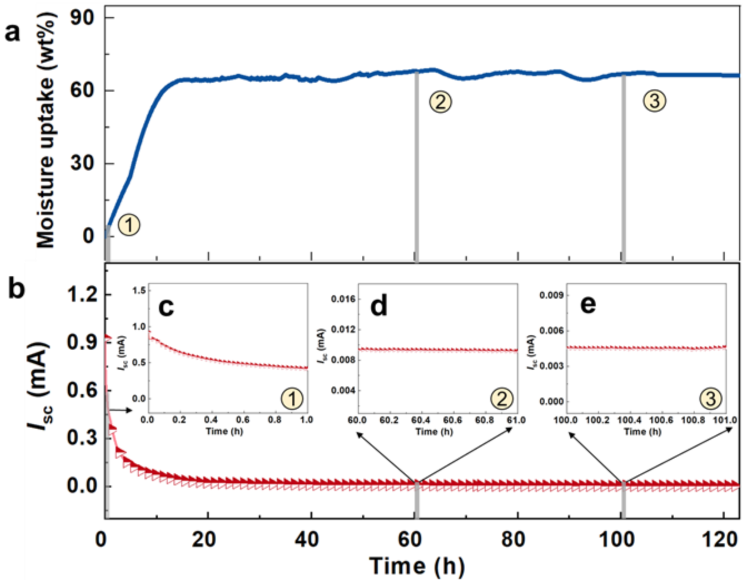

Supplementary Figure 14. Moisture uptake capability and current output of MEG versus time synchronously. (a) The mass increase of MEG by moisture adsorption versus time under 80% RH and room temperature. (b) The concurrent current output of MEG versus time at the same condition. (c-e) The tested current output of MEG at different time slot.

Supplementary Figure 14a clearly demonstrates that moisture absorption of MEG increases quickly at the first 16 hours and then slowly reaches a platform about 68 wt%, followed by a small fluctuation for a long term. That means that the moisture absorption of MEG reaches a saturation state with moisture adsorption and desorption dynamically. At the same time, a

continuous current output generates over 100 hours (Supplementary Figure 14b), which associates well with the moisture adsorption process. The continuous adsorption of water molecules couples with the ion dissociation process in the MEG, leading to a continuous current output even after reaching absorption saturation state despite a gradually decayed current. The possible reason lies in the ion concentration is expected to establish an equilibrium over a long period. [12] It well demonstrates that moisture absorption process is directly related to electricity generation of MEG.

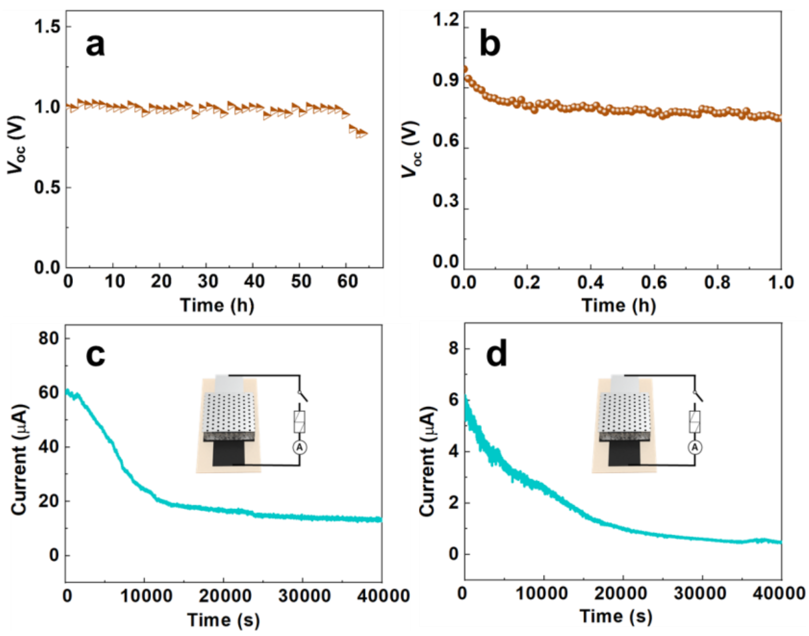

Supplementary Figure 15. (a, c) The V_{oc} curves and current output of MEG devices based on AlgNa hydrogel over time under an open environment and room temperature, respectively. (b, d) The V_{oc} curves and current output of MEG devices based on PVA hydrogel with 1 kΩ external resistor under the evolution of time at 70% RH and room temperature, respectively. The insert shows the circuit diagram of MEG with one resistor.

To verify the sustained durability in power output for AlgCa > AlgNa > PVA experimentally, we directly prepared MEGs with water-soluble PVA hydrogel and AlgNa hydrogel, separately. While for AlgCa, it is insoluble in water and cannot be directly prepared. [13] As an alternative to investigate the effect of AlgCa, PVA-AlgNa based hydrogel in our work was applied, where AlgCa ionically cross-linked network is formed as shown in Supplementary Figure 1. The sustained voltage and current are compared as shown in Supplementary Figure 15.

For the voltage output, Supplementary Figure 15a shows AlgNa based MEG delivers a continuous DC V_{oc} of about 1.0 V for more than 60 hours. While PVA based MEG only exports a stable DC V_{oc} of about 0.8 V after a drop from 1.0 V within 20 minutes (Supplementary Figure 15b). Compared to PVA-AlgNa based MEG (90 hours), it can see the sustainability of V_{oc} follows the order: AlgCa > AlgNa > PVA. The sustained durability in current was also tested by loading with 1 kΩ external resistor. PVA-AlgNa based MEG presents a steady current output for about 17 hours (Fig. 1e). In contrast, the current of AlgNa based MEG reduces by 33% within 2 hours while that of PVA based MEG drops by 50% at the same time (Supplementary Figure 15c and d). The current sustainability also follows the same order. The above results demonstrate that the sustained durability in electrical energy output as follows: AlgCa > AlgNa > PVA, which well verifies the DFT simulation results.

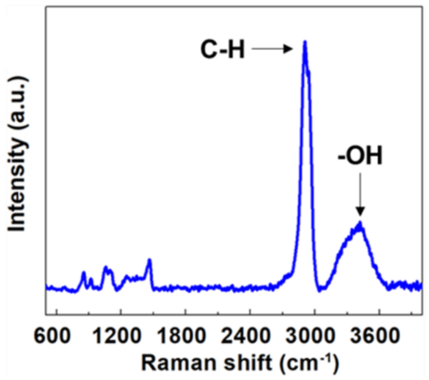

Supplementary Figure 16. Raman spectrum of the PVA-AlgNa based hydrogel.

The Raman result in Supplementary Figure 16 shows that C-H bonds of stretching vibration area within 2800-3000 cm^{-1} and O-H bonds of stretching vibration area within 3050-3650 cm^{-1}, where Raman band ratio O-H bonds/C-H bonds can be used to track the moisture absorption as shown in Supplementary Figure 16 and S17. The FTIR spectrum (Supplementary Figure 1) shows that 3279 cm^{-1} is assigned as the O-H stretching absorption peak of PVA-AlgNa based hydrogel, which is closely related to the water absorption process. Thus the time-dependent FTIR was also used to track water absorption and diffusion process.

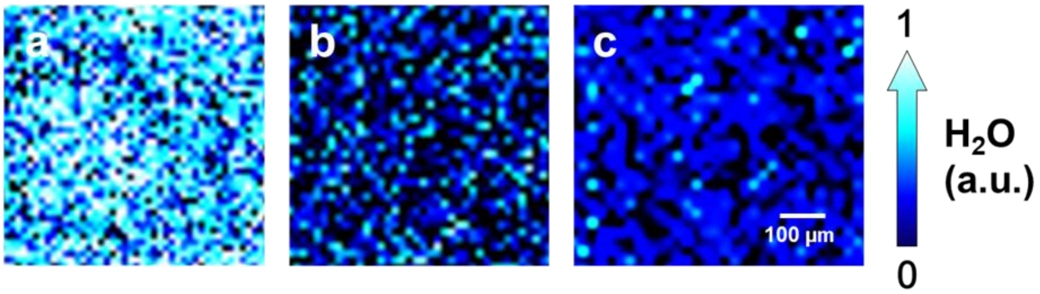

Supplementary Figure 17. 2D Raman mapping. (a) The moisture capturing by top surface of PVA-AlgNa based hydrogel after exposing in air for 45 min. (b) The moisture capturing by bottom surface of PVA-AlgNa based hydrogel with the same exposure time. (c) The moisture capturing by top surface of PVA after exposed in air for 45 min.

In-situ Raman spectroscopy mapping was employed to track the sorption path of water molecules on hydrogel surfaces by detecting Raman band ratio O-H bond/C-H bond. Supplementary Figure 17 shows that the top surface of PVA-AlgNa based hydrogel has captured more water molecules than the bottom surface at the same exposure time, implying the existence of a built-in water gradient. Besides, the control sample PVA hydrogel shows low moisture intake capability than the PVA-AlgNa based hydrogel (Fig S17c).

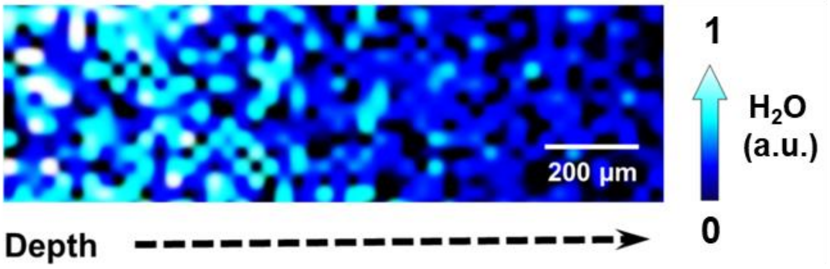

Supplementary Figure 18. Cross-sectional 2D Raman mapping of PVA-AlgNa based hydrogel with the depth after one week.

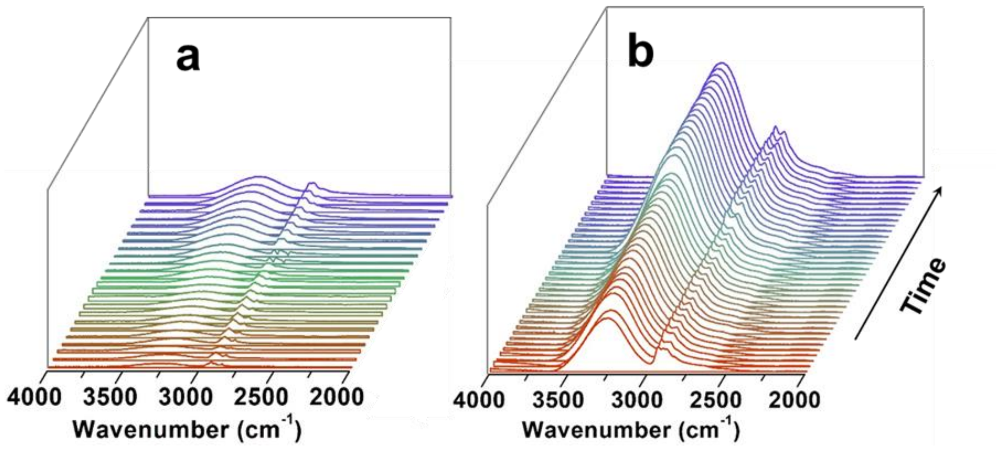

Supplementary Figure 19. In-situ FTIR spectrum tracking once the samples are exposed in the atmosphere (65% RH) versus time for PVA (a) and PVA-AlgNa based hydrogel (b).

There seems to be an obvious increase in ν (OH) band intensities for the MEG compared to the control sample (Supplementary Figure 19), suggesting a stronger moisture capturing ability of the MEG.

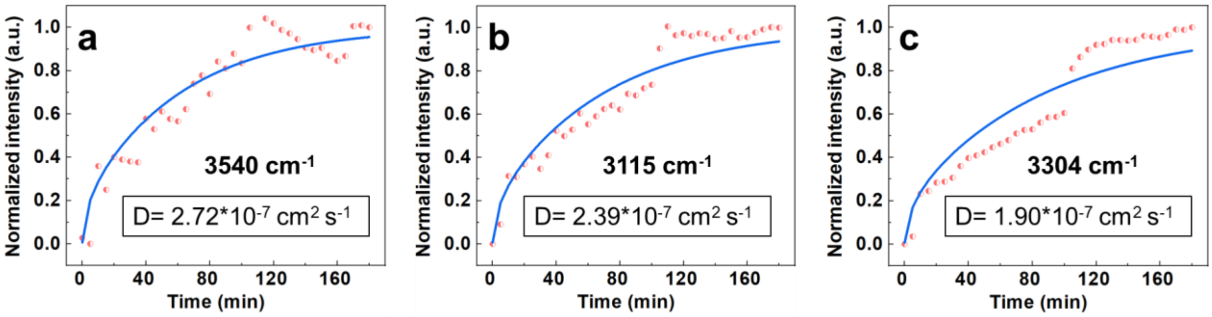

Supplementary Figure 20. The integrated intensities of the three decoupled peaks versus time, (a) 3540 cm^{-1}; (b) 3115 cm^{-1}, (c) 3304 cm^{-1}.

To quantitatively analyze the water molecules diffusion ability of hydrogel, we further extract quantitative information from the 1D-FTIR profiles by integrating the intensities of the 2D-FTIR patterns. Hence, the intensities of three decoupled peaks (3540, 3115, 3304 cm^{-1}) with the time were obtained. Furthermore, equation 1 is given to estimate the effective diffusion coefficient of water from FTIR spectra based on the Fickian diffusion. [14]

$$\frac{A_t}{A_\infty} = 1 - \frac{8\gamma}{\pi[1-\exp(-2\gamma L)]} \times \sum_{n=0}^{\infty} \left\{ \frac{\exp(g)\,[f exp(-2\gamma L) + (-1)^n (2\gamma)]}{(2n+1)(4\gamma^2 + f^2)} \right\}$$

(1)

Where

$$g = \frac{-D(2n+1)^2\pi^2 t}{4L^2}, f = \frac{(2n+1)\pi}{2L}$$

In equation 1, A_t is the band absorbance of the FTIR spectra at time t, A_∞ is the band absorbance at equilibrium, γ is the penetration depth of the evanescent wave, L is the thickness of the polymer membrane (invariable), and D is the diffusion coefficient. The different diffusion coefficients of different states of water can be calculated by a nonlinear curve fitting[15] to equation 1 from the variation of the three decoupled peaks (3540, 3115, 3304 cm^{-1}) versus time.

The results are shown in Supplementary Figure 20. The D value for three peaks also follows the order: 3540 > 3115 > 3304 cm^{-1}, which agrees well with the results of 2D-FTIR spectroscopy. That means weak bound water diffuses in the fastest way, while ion water cluster diffuses in the slowest way due to intense attraction force by AlgCa/Na network.

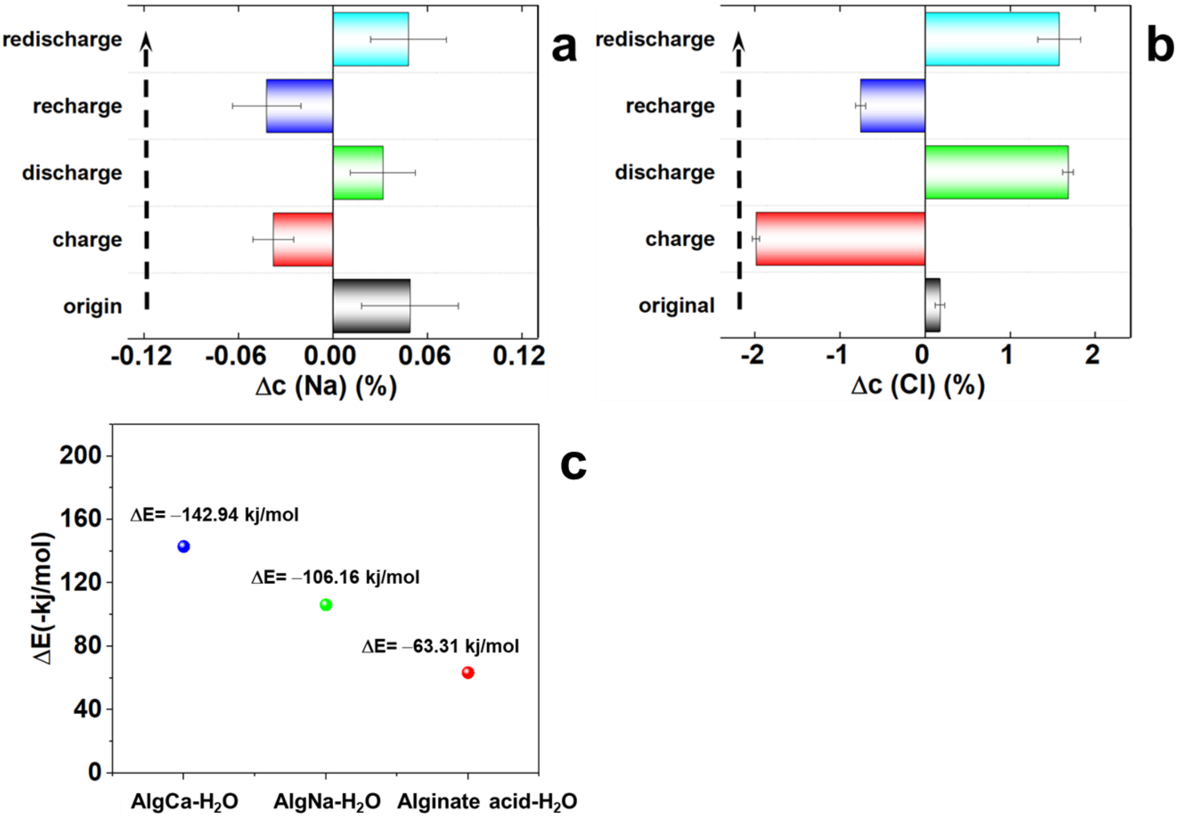

Supplementary Figure 21. Chemical component characterization of detective Na^+ ions (a) and Cl^- ions (b) variation between the two surfaces of MEG in different states. The Δc(ion) is defined as the ion element content at the top surface minus the content at the bottom surface of MEG. Data represent the mean ± standard deviation (n = 4). (c) Interaction region indicator maps and the corresponding interaction energies (kj/mol) of AlgCa-H_2O, AlgNa-H_2O, Alginate acid-H_2O. The sign(λ_2)ρ is mapped on the isosurfaces.

Supplementary Figure 21a and b show the Na and Cl ions transport from top to bottom during charging, and vice versa. Supplementary Figure 21c shows that the absorption energy of Alginate-H_2O is about −63.31 kj/mol by DFT calculation, which is smaller than that of AlgNa-H_2O (−106.16 kj/mol). The weaker interaction between Alginate acid and H_2O leads to inferior chemical energy conversion. Therefore, less protons are dissociated from Alg chains and MEG using Alginate acid generates inferior current. In clear contrast, the absorption energy (−142.94 kj/mol) of AlgCa-H_2O is the strongest, enabling sufficient chemical conversion energy to trigger Ca ions to dissociate from the polymer chains and thus rich Ca ions transport is observed. As a positive result, MEGs based on AlgNa/Ca hybrid hydrogels output large current. The electric output is well consistent with the absorption energy of hybrid hydrogels. It further verifies the moisture is key to the electric generation for MEGs.

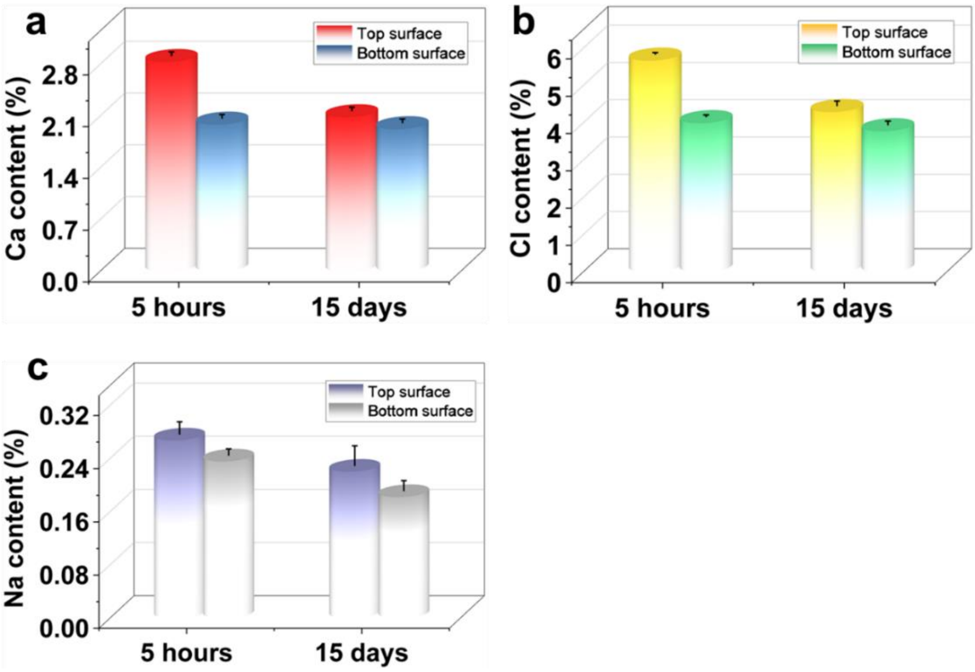

Supplementary Figure 22. The ion distribution between top and bottom surface for MEG suffering from 5 hours' and 15 days' short-circuit treatment. (a) Ca element. (b) Cl element. (c)

Na element. Data represent the mean ± standard deviation (n = 4).

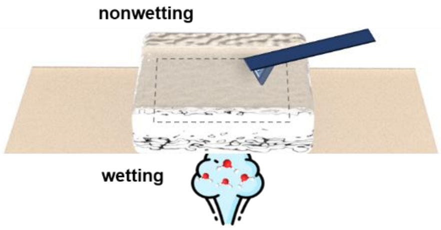

Supplementary Figure 23. Schematic plot of the setup for the KPFM test. A piece of hydrogel absorbs moisture from its bottom side and the upper side is covered without wetting. The KPFM probe measures the nonwetting side with the time.

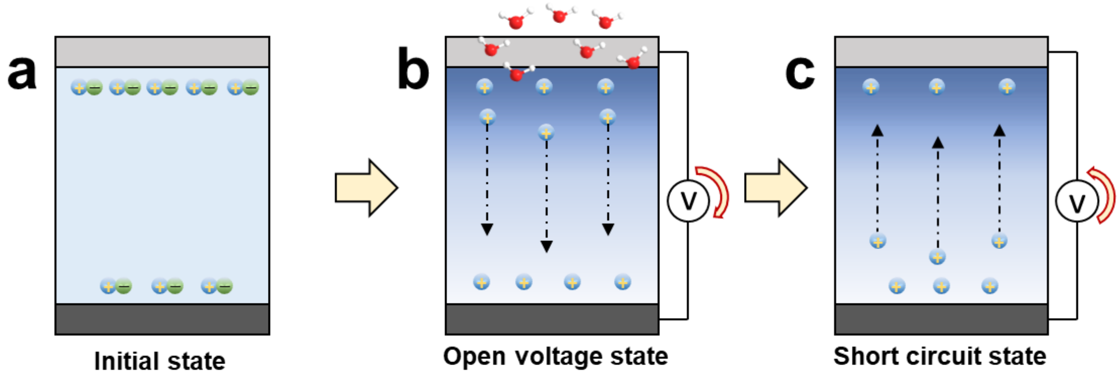

Supplementary Figure 24. Schematic plot of proposed working mechanism of MEG. (a) The initial state. Ions pair are distributed along the thickness direction due to the weak internal electric field of asymmetrical structure at the initial state. (b) Open voltage stage. After absorbing water molecules from air, positive ions transport from the top to the bottom side, resulting in a large open-circuit voltage. (c) Short circuit stage. The reverse migration of ions is observed with short-circuit treatment.

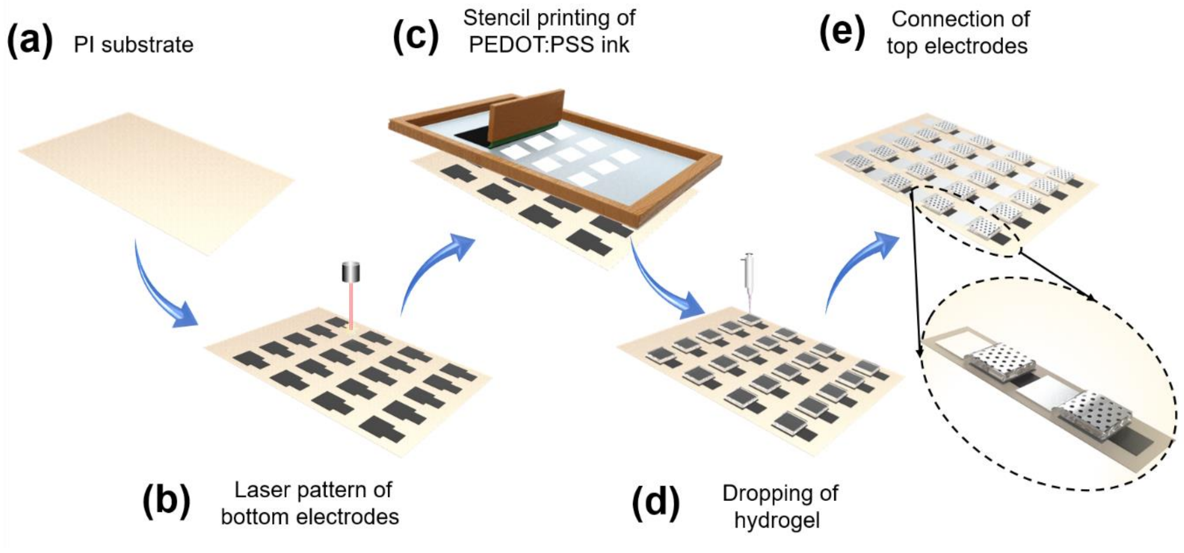

Supplementary Figure 25. The fabrication process diagram of large-scale integration of MEG devices. (a) Preparation of PI substrate. (b) Laser printing of bottom electrodes with optimized laser power and writing speed. (c) Stencil printing of PEDOT: PSS ink on bottom electrode as carrier transport-assisting layer. (d) Dripping well-prepared hydrogel sequentially. (e) Placing and adhering top electrodes on the top of the hydrogel by end-to-end connection. The inset is the structure of enlarged 2-serial MEG units. The details can be found in the experimental section.

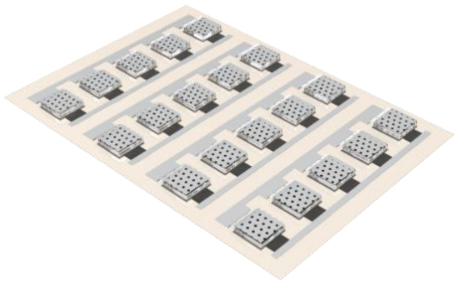

Supplementary Figure 26. Scheme of large-scale integration of parallel MEG bank.

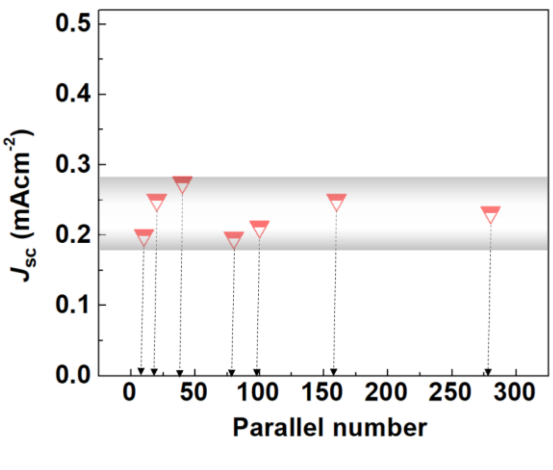

Supplementary Figure 27. The current density for the large-scale MEG array.

Supplementary Table 1. Surface area in each ESP range on the vdW surface for PVA.

Begin	End	Area	%
-80	-60	0.00	0.00
-60	-40	0.00	0.00
-40	-20	8.47	6.31
-20	0	41.52	30.92
0	20	78.21	58.26
20	40	5.91	4.40
40	60	0.14	0.11
60	80	0.00	0.00
80	100	0.00	0.00
100	120	0.00	0.00
120	140	0.00	0.00
140	160	0.00	0.00
160	180	0.00	0.00
Sum:		134.25	100.00

Supplementary Table 2. Surface area in each ESP range on the vdW surface for AlgNa.

Begin	End	Area	%
-80.00	-58.33	0.00	0.00
-58.33	-36.67	23.90	7.29
-36.67	-15.00	105.96	32.34
-15.00	6.67	120.57	36.80
6.67	28.33	44.52	13.59
28.33	50.00	8.56	2.61
50.00	71.67	4.59	1.40
71.67	93.33	6.38	1.95
93.33	115.00	6.36	1.94
115.00	136.67	6.81	2.08
136.67	158.33	0.00	0.00
158.33	180.00	0.00	0.00
Sum:		327.63	100.00

Supplementary Table 3. Surface area in each ESP range on the vdW surface for AlgCa.

Begin	End	Area	%
-80	-60	24.93	7.74
-60	-40	37.52	11.65
-40	-20	67.89	21.07
-20	0	82.23	25.52
0	20	56.69	17.59
20	40	15.46	4.80
40	60	7.87	2.44
60	80	6.42	1.99
80	100	2.95	0.92
100	120	2.41	0.75
120	140	2.44	0.76
140	160	4.11	1.28
160	180	9.73	3.02
Sum:		320.65	99.52

Supplementary Table 4. The performance comparison of current moisture electric generators.

Materials	Ionic conductivity ($S\ cm^{-1}$)	Moisture uptake capability (wt%)	Current ($\mu A\ cm^{-2}$)	Voltage (V)	Power ($\mu W\ cm^{-2}$)	RH (%)	Ref.
PSSA/PDDA	~$3*10^{-3}$	30	4	1	5.52	75	2
Carbon black-sodium dodecyl benzene sulfonate	$8*10^{-5}$	150	7	0.7	226 $\mu W\ g^{-1}$	80	16
PSSA/R film	$3.1*10^{-6}$	109.9	160	0.82	88	50	17
PVA-PA-Gly gel	$1.76*10^{-4}$	30.0	240	0.8	35	80	18
G. sulfurreducens PCA film	$1.6*10^{-3}$	6.0	13	0.35	5.1	90	19
GO/PVA	$6*10^{-7}$	n.a.	92.8	0.85	n.a.	55	21
PSS/PVA textile	$3.75*10^{-7}$	n.a.	1.5	1	0.1	80	22
LiCl@ cellulon-Carbon black@ cellulon paper	$1.5*10^{-6}$	30	3	0.78	0.7	50	23
SA-SiO_2-RGO	$1.1*10^{-3}$	240	100	0.5	12	100	24
Waste activated sludge	$1.8*10^{-3}$	15.5	2.98	0.45	5.24	90	25
CS/SWNTs/PVA/CNF aerogel	$2.6*10^{-3}$	600	117	1.45	32.59	80	26

PSSA-kuromanin (chloride) film	0.1	227	300	0.8	n.a.	70	27
Sulfonate-polyaniline-bifunctionalized lignin	$7.25*10^{-2}$	40	0.125	0.28	44.5 W kg^{-1}	99	28
Protein nanowire	$1.2*10^{-6}$	27	40	0.5	5	50	29
Asymmetric GO	$3*10^{-6}$	n.a.	0.6	0.45	2.02	25	30
PVA-AlgNa based hydrogel	$1.02*10^{-3}$	68	408	1.3	110	80	this work

Supplementary References

1. X. Jing, H. Li, H.-Y. Mi, P.-Y. Feng, X. Tao, Y. Liu, C. Liu and C. Shen. Enhancing the performance of a stretchable and transparent triboelectric nanogenerator by optimizing the hydrogel ionic electrode property, *ACS Appl. Mater. Interfaces* **12**, 23474-23483, (2020).
2. H. Wang, Y. Sun, T. He, Y. Huang, H. Cheng, C. Li, D. Xie, P. Yang, Y. Zhang and L. Qu. Bilayer of polyelectrolyte films for spontaneous power generation in air up to an integrated 1,000 V output, *Nat. Nanotechnol.* **16**, 811-819, (2021).
3. R. Zhu, Y. Zhu, L. Hu, P. Guan, D. Su, S. Zhang, C. Liu, Z. Feng, G. Hu and F. Chen. Lab free protein-based moisture electric generators with a high electric output, *Energ. Environ. Sci.* **16**, 2338-2345, (2023).
4. T. Xu, X. Ding, Y. Huang, C. Shao, L. Song, X. Gao, Z. Zhang and L. Qu. An efficient polymer moist-electric generator, *Energ. Environ. Sci.* **12**, 972-978, (2019).
5. Q. Li, Y. Qin, D. Cheng, M. Cheng, H. Zhao, L. Li, S. Qu, J. Tan and J. Ding. Moist-Electric Generator with Efficient Output and Scalable Integration Based on Carbonized Polymer Dot and Liquid Metal Active Electrode, *Adv. Funct. Mater.* **33**, 2211013, (2023).
6. F. Zhao, Y. Liang, H. Cheng, L. Jiang and L. Qu. Highly efficient moisture-enabled electricity generation from graphene oxide frameworks, *Energ. Environ. Sci.* **9**, 912-916, (2016).
7. H. Wang, H. Wang, Y. Wang, X. Su, C. Wang, M. Zhang, M. Jian, K. Xia, X. Liang and H. Lu. Laser writing of janus graphene/kevlar textile for intelligent protective clothing, *ACS nano* **14**, 3219-3226, (2020).
8. J. Lin, Z. Peng, Y. Liu, F. Ruiz-Zepeda, R. Ye, E. L. Samuel, M. J. Yacaman, B. I. Yakobson and J. M. Tour. Laser-induced porous graphene films from commercial polymers, *Nat. Commun.* **5**, 5714, (2014).
9. X. Liu, J. Tan, J. Fu, R. Yuan, H. Wen and C. Zhang. Facile synthesis of nanosized lithium-ion-conducting solid electrolyte Li1. 4Al0. 4Ti1. 6 (PO4) 3 and its mechanical nanocomposites with LiMn2O4 for enhanced cyclic performance in lithium ion batteries, *ACS Appl. Mater. Interfaces* **9**, 11696-11703, (2017).
10. K. H. Park, D. Y. Oh, Y. E. Choi, Y. J. Nam, L. Han, J. Y. Kim, H. Xin, F. Lin, S. M. Oh and Y. S. Jung. Solution-processable glass LiI-Li4SnS4 superionic conductors for all-solid-state Li-ion batteries, *Adv. Mater.* **28**, 1874-1883, (2016).
11. B.-A. Mei, O. Munteshari, J. Lau, B. Dunn and L. Pilon. Physical interpretations of Nyquist plots for

EDLC electrodes and devices, *The Journal of Physical Chemistry C* **122**, 194-206, (2018).
12. Y. Huang, H. Cheng, C. Yang, H. Yao, C. Li and L. Qu. All-region-applicable, continuous power supply of graphene oxide composite, *Energ. Environ. Sci.* **12**, 1848-1856, (2019).
13. Y. Qin. Alginate fibres: an overview of the production processes and applications in wound management, *Polym. Int.* **57**, 171-180, (2008).
14. B. Tang, P. Wu and H. Siesler. In situ study of diffusion and interaction of water and mono-or divalent anions in a positively charged membrane using two-dimensional correlation FT-IR/attenuated total reflection spectroscopy, *The Journal of Physical Chemistry B* **112**, 2880-2887, (2008).
15. M. Liu, P. Wu, Y. Ding, G. Chen and S. Li. Two-dimensional (2D) ATR− FTIR spectroscopic study on water diffusion in cured epoxy resins, *Macromolecules* **35**, 5500-5507, (2002).
16. Y. Zhang, S. Guo, Z. G. Yu, H. Qu, W. Sun, J. Yang, L. Suresh, X. Zhang, J. J. Koh and S. C. Tan. An Asymmetric Hygroscopic Structure for Moisture-Driven Hygro-Ionic Electricity Generation and Storage, *Adv. Mater.*, 2201228, (2022).
17. J. Bai, Y. Huang, H. Wang, T. Guang, Q. Liao, H. Cheng, S. Deng, Q. Li, Z. Shuai and L. Qu. Sunlight-Coordinated High-Performance Moisture Power in Natural Conditions, *Adv. Mater.* **34**, 2103897, (2022).
18. S. Yang, X. Tao, W. Chen, J. Mao, H. Luo, S. Lin, L. Zhang and J. Hao. Ionic Hydrogel for Efficient and Scalable Moisture-Electric Generation, *Adv. Mater.*, 2200693, (2022).
19. G. Ren, Q. Hu, J. Ye, X. Liu, S. Zhou and Z. He. Hydrovoltaic effect of microbial films enables highly efficient and sustainable electricity generation from ambient humidity, *Chem. Eng. J.* **441**, 135921, (2022).
20. D. Lv, S. Zheng, C. Cao, K. Li, L. Ai, X. Li, Z. Yang, Z. Xu and X. Yao. Defect-enhanced selective ion transport in an ionic nanocomposite for efficient energy harvesting from moisture, *Energ. Environ. Sci.* **15**, 2601-2609, (2022).
21. R. Zhu, Y. Zhu, F. Chen, R. Patterson, Y. Zhou, T. Wan, L. Hu, T. Wu, R. Joshi and M. Li. Boosting moisture induced electricity generation from graphene oxide through engineering oxygen-based functional groups, *Nano Energy* **94**, 106942, (2022).
22. W. He, H. Wang, Y. Huang, T. He, F. Chi, H. Cheng, D. Liu, L. Dai and L. Qu. Textile-based moisture power generator with dual asymmetric structure and high flexibility for wearable applications, *Nano Energy* **95**, 107017, (2022).
23. J. Tan, S. Fang, Z. Zhang, J. Yin, L. Li, X. Wang and W. Guo. Self-sustained electricity generator driven by the compatible integration of ambient moisture adsorption and evaporation, *Nat. Commun.* **13**, 1-8, (2022).
24. H. Wang, T. He, X. Hao, Y. Huang, H. Yao, F. Liu, H. Cheng and L. Qu. Moisture adsorption-desorption full cycle power generation, *Nat. Commun.* **13**, 1-11, (2022).
25. J. Liu, X. Cai, Y. Wang, G. Ren, L. Zhou, M. Mahmoud, S. Zhou and Y. Yuan. Moisture-induced electrical power generation with waste activated sludge, *Chem. Eng. J.*, 144868, (2023).
26. X. Zhang, Z. Dai, J. Chen, X. Chen, X. Lin, S. Yang, K. Wu, Q. Fu and H. Deng. Double-gradient-structured composite aerogels for ultra-high-performance moisture energy harvesting, *Energ. Environ. Sci.*, (2023).
27. J. Bai, Q. Liao, H. Yao, T. Guang, T. He, H. Cheng and L. Qu. Self-induced interface enhanced moisture-harvesting and light-trapping toward high performance electric power generation, *Energ. Environ. Sci.*, (2023).
28. H. Zheng, A. Zhou, Y. Li, X. Chen, Y. Chen, Y. Xu, Y. Li, H. Ge and X. Ning. A sandwich-like flexible nanofiber device boosts moisture induced electricity generation for power supply and multiple sensing

applications, *Nano Energy* **113**, 108529, (2023).

29. X. Liu, H. Gao, J. E. Ward, X. Liu, B. Yin, T. Fu, J. Chen, D. R. Lovley and J. Yao. Power generation from ambient humidity using protein nanowires, *Nature* **578**, 550-554, (2020).

30. H. Cheng, Y. Huang, F. Zhao, C. Yang, P. Zhang, L. Jiang, G. Shi and L. Qu. Spontaneous power source in ambient air of a well-directionally reduced graphene oxide bulk, *Energ. Environ. Sci.* **11**, 2839-2845, (2018).
