# Supplementary material for: Green moisture-electric generator based on supramolecular hydrogel with tens of milliamp electricity toward practical applications
Source: Nat Commun. 2024 Apr 18;15:3329. doi: 10.1038/s41467-024-47652-3 (PMC11026426; doi:10.1038/s41467-024-47652-3)
Supplement: Supplementary file 4 — Description of Additional Supplementary Files [file 41467_2024_47652_MOESM4_ESM.pdf]

Description of Additional Supplementary Files

File Name: Supplementary Movie 1

Description: A MEG shirt worn on a sportsman charges a smart watch.

File Name: Supplementary Movie 2

Description: A lamp bulb of 2.5 W is illuminated continuously by a largescale MEGs bank with a 10*24 in parallel*serial combination.

File Name: Supplementary Movie 3

Description: A LCD clock runs one month driven by a two-serial MEG bank.
